# Identification of several senescence‐associated genes signature in head and neck squamous cell carcinoma

**DOI:** 10.1002/jcla.24555

**Published:** 2022-06-12

**Authors:** Jian Wang, Chong‐Chang Zhou, Hong‐Cun Sun, Qun Li, Jian‐Dao Hu, Tao Jiang, Shao Zhou

**Affiliations:** ^1^ Department of Otorhinolaryngology Head and Neck Surgery The Affiliated People's Hospital of Ningbo University Ningbo China; ^2^ Department of Otorhinolaryngology Head and Neck Surgery Ningbo Medical Center Lihuili Hospital Ningbo China

**Keywords:** GEO, overall survival, senescence‐associated genes signature, head and neck squamous cell carcinoma, prognosis, TCGA

## Abstract

**Background:**

As one of the core aging processes, cellular senescence is associated with tumorigenesis, growth, and immune modulation in cancers. Nevertheless, the prognosis of senescence‐associated genes (SAGs) signature in head and neck squamous cell carcinoma (HNSCC) remains to be further evaluated.

**Methods:**

The transcriptome and corresponding clinical datasets of SAGs in patients with HNSCC were downloaded from public databases. A new prognostic SAGs signature was established with least absolute shrinkage and selection operator discussion. Patients with HNSCC were fallen into two risk groups based on each sample's risk mark and the cutoff point. The survival analysis was extended to determine the predictive accuracy of the SAGs signature. Furthermore, the evaluation of SAGs signature was made according to clinicopathological characteristics, survival state, the infiltration of inflammatory cells, and efficacy of immunotherapy.

**Results:**

41 SAGs were recognized and adopted to establish the forecast signature. The survival analysis indicated that patients with HNSCC in the high‐senescent score group had significantly reduced overall survival compared with those in the low‐senescent score group. It was certified that the risk score of SAGs signature was a separate predicting agent for HNSCC applying Cox regression analysis. According to functional analysis, some immune‐associated pathways were increased in the low‐senescent score group significantly. High‐senescent score group was correlated with poor clinicopathological characteristics, given less the infiltration of inflammatory cells state and worse immunotherapeutic effect.

**Conclusion:**

A new SAG signature predicting result and response to immunotherapy of HNSCC was identified. Cellular senescence may be a hidden target for HNSCC.

## Graphical abstract

In this study, HNSCC clinical data and senescence‐associated genes data were extracted from the public database. Prognostic differential genes were screened and senescence‐associated genes signature was constructed. The survival analysis indicated that patients with HNSCC in the high senescent score group had significantly reduced overall survival compared with those in the low senescent score group. Some immune‐associated pathways were increased in the low senescent score group significantly. High senescent score group was correlated with poor clinicopathological characteristics, given less the infiltration of inflammatory cells state and worse immunotherapeutic effect. Gemcitabine was more sensitive in patients with high senescent risk.

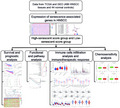

## 1 INTRODUCTION

Head and neck squamous cell carcinoma (HNSCC) ranks the sixth in terms of frequent cancer globally; there are about 0.6 million new cases diagnosed each year.^1^ The primary treatment of HNSCC rely on the clinical phase including operation, radiotherapy, chemotherapy and immunotherapy. Treatments for a persistently high mortality rate in advanced HNSCC patients are limited; the 5‐year total survival rate is only 63%.^2^ For improving the prognosis results of HNSCC patients, steady prognostic signatures shall be identified.

**FIGURE 1:**
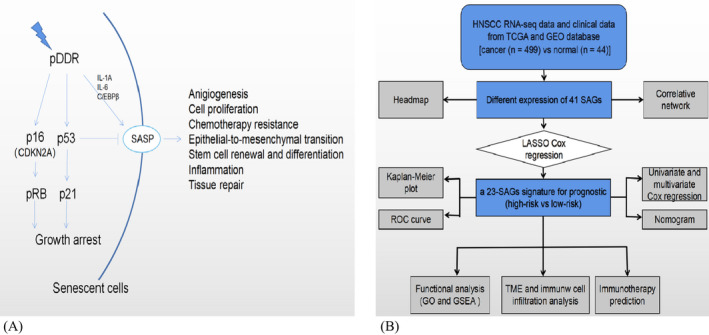
(A) Schematic diagram of the cellular senescence process. (B) The flow chart of this study. Abbreviations: pDDR, Persistent DNA damage response; SASP, senescence‐associated secretory phenotype; HNSCC, Head and neck squamous cell carcinoma; TCGA, the cancer genome atlas; GEO, The Gene Expression Omnibus; SAGs, Senescence‐Related Genes; LASSO, least absolute shrinkage and selection operator; ROC, receiver operating characteristic; TME, tumor microenvironment; GO, Gene Ontology; GSEA, Gene set enrichment analysis

Cellular senescence, one of the core aging processes, has the characteristics such as an eternal cell cycle arrest.^3^ It is considered to be a response to injury inside and outside the cell. Nevertheless, the relationship of cell senescence and tumor, particularly in HNSCC, is complicated and poorly interpreted as yet. It has been deemed that cellular senescence is a double‐edged sword during tumorigenesis and growth. In one aspect, aging cells go into eternal cell cycle arrest and be cleared by the human immune system, which inhibits cancer growth in prophase.^4^ On the other hand, when immune system cannot effectively remove aging cells, this accumulation of senescence cells facilitates the senescence‐related secretory phenotype (SASP),^5^ which changes immune microenvironment and remodels tissue by releasing growth elements, cytokines, enzymes, and extracellular matrix (ECM) components, leading to tumorigenesis and development.^3^ In the current studies, both an interior persistent DDR and extrinsic stress promote the progress of tumorigenesis and tumor cell evasion from immunosurveillance, bringing worse prognosis in HNSCC patients (Figure 1A)^6^, ^7^.

For assessing the relationship between cellular senescence and prognosis in HNSCC in an integrated way, we constructed a new risk model on basis of differentially expressed and prognostic senescence‐associated genes (SAGs) and dug their hidden value as sibylline biomarkers for prognosis and immunotherapy response from databases. Next, the SAGs scoring model can forecast total survival of HNSCC patients and guide clinical treatment by calculating the total risk mark. In the end, hidden measures for treating HNSCC were identified by analyzing the association between cellular senescence, prognosis, the immune microenvironment, and the response to immunotherapy.

## 2 MATERIALS AND METHODS

### 2.1 Data sources and processing

CellAge (Avelar, et al., 2020) (https://genomics.senescence.info/cells/) provided the list of genes with manually gathered information of human genes related to cellular senescence.^8^ There were 279 genes in this research. The Cancer Genome Atlas (TCGA, https://portal.gdc.cancer.gov) website offered clinical data and transcriptional profiles of 499 HNSCC patients and 44 normal tissues as the training cohort. The GSE65858 with the sample size was enrolled from The Gene Expression Omnibus (GEO, http://www.ncbi.nlm.nih.gov/geo) as the validation cohorts.

### 2.2 Differential gene expression analysis

The differentially expressed genes (DEGs) between tumor samples (*n* = 499) and normal tissues (*n* = 44) was performed with R package “limma”. Pearson associations >0.30 and *p* < 0.05 were adopted to set up the expression association network of SAGs in HNSCC.

### 2.3 Establishment of a prognostic SAGs signature

Differentially expressed SAGs were screened by performing univariate Cox analysis applying R package “survival” based on the thresholds of *p* < 0.01. We identified and developed the optimal prognostic SAGs signature applying the “glmnet” R package by adopting the least absolute shrinkage and selection operator (LASSO) Cox regression discussion. The calculation of risk marks of HNSCC samples was made based on related regression parameters and expression level of each gene: Risk mark = $\sum_{i=0}^{n}\text{coefficient}\;$ x different expression levels of SAGs. Furthermore, 499 HNSCC patients were fallen into high‐ and low‐risk groups on basis of the average value of risk marks. The receiver operating characteristic (ROC) curve of 1‐, 3‐, and 5‐years was constructed to assess the effectiveness of the SAGs signature model. The comparison of total survival diversities between two risk groups was made by performing Kaplan–Meier survival curves. If the SAGs signature was assessed as a hidden independent prognostic predictor of HNSCC patients applying univariate and multivariate Cox regression explorations. A nomogram was established for survival prediction by including age, N stage, T stage, gender, grade, and risk score. The precision of the nomogram was assessed with the calibration curves. Chi‐square tests and Wilcoxon signed‐rank tests were adopted to assess clinicopathological elements.

### 2.4 Functional enrichment and pathway analyses

On basis of DEGs, Gene Ontology (GO) analyses was made with a modified *p* < 0.05. Gene set enrichment analyses (GSEA, version 4.1.0) between two risk groups was utilized to find a possible molecular basis.

### 2.5 Immune cell infiltration and immunotherapy

We calculated the immune mark, stromal mark and estimate mark of each HNSCC sample applying the ESTIMATE algorithm.^9^ The cibersort method was adopted to find the fraction of immune‐related cell types.^10^ The different infiltrating levels of immune cell between two risk groups were calculated. The single‐sample gene set enrichment analysis (ssGSEA) was adopted to obtain the immune‐related pathway. The high‐senescent score and low‐senescent score groups were compared in immune checkpoint inhibitor (ICI)‐associated genes. Immunotherapy response was evaluated to explore the value of ICI treatments for HNSCC by The Cancer Immunome Atlas (TCIA, https://tcia.at/) website^10^.

### 2.6 Chemotherapeutic drug sensitivity analysis

In order to study the sensitivity of high‐ and low‐senescent score groups to chemotherapy drugs, the “pRRophetic” package was used to evaluate HNSCC patients' half‐maximum inhibitory concentration (IC50) of four conventional chemotherapy drugs (paclitaxel, gemcitabine, docetaxel, and cisplatin).

### 2.7 Statistical analysis

All statistical analysis and figures were performed applying R language (version 4.1.0). The Chi‐square test and Wilcoxon signed‐rank tests were employed for classified and constant variables data. Pearson correlation analysis was adopted for association assessment. *p* < 0.05 meant statistical significance.

## 3 RESULTS

### 3.1 Recognition of differentially expressed senescence‐associated genes in HNSCC

The comparison of the expression of 256 SAGs was made between 499 HNSCC tissues and 44 non‐tumor samples retrieved from TCGA and GEO after excluding the 23 samples of mismatched data. We identified 193 SAGs greatly differentially expressed among HNSCC patients (*p* < 0.05).

### 3.2 Construction of a prognostic SAG signature

**FIGURE 2:**
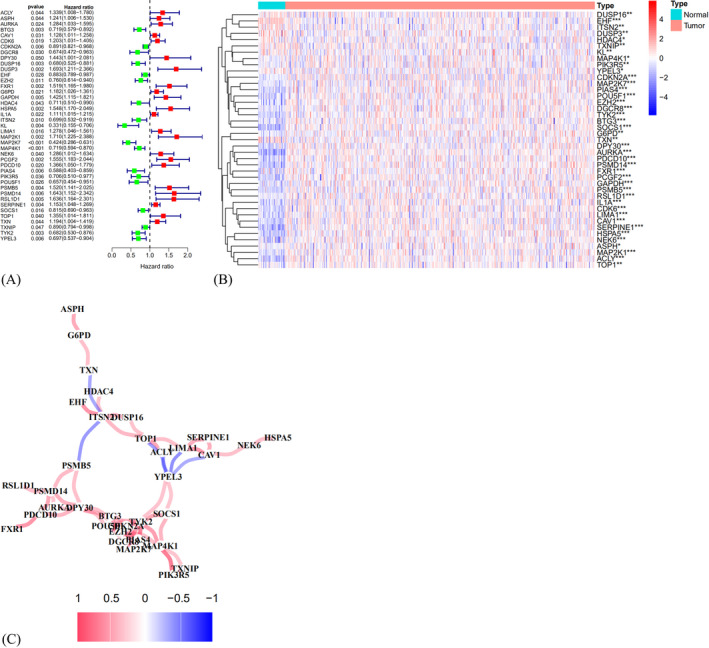
Construction of the prognostic SAGs and the expression landscape of 41 prognostic SAGs in HNSCC patients. (A) The forest plot of 41 prognostic SAGs associated with overall survival was shown using univariate Cox regression analysis (*p* < 0.2). (B) The heatmap of 41 prognostic SAGs between 499 HNSCC tissues and 44 adjacent normal tissues (*p < 0.05; **p < 0.01; ***p < 0.001). (C) The correlation network of the SAGs (red line: positive correlation; blue line: negative correlation. The depth of the colors represents the degrees of correlation)

**FIGURE 3:**
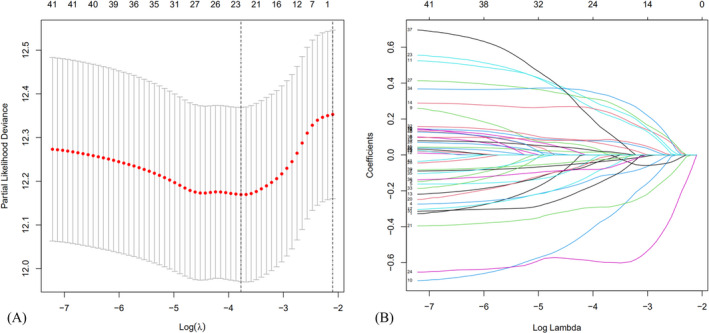
(A) Optimal parameter (λ) selected in the LASSO Cox regression model based on the minimum criteria. (B) The LASSO coefficient of the senescence‐related signature

As shown in Figure 2A, 41 prognostic SAGs were recognized with univariate Cox regression analysis (Table 1). The heatmap was set up in Figure 2B (red means high expression and green low expression). Figure 2C shows the expression association network of the SAGs in HNSCC (red displays positive association and blue negative association). Based on best value of λ and coefficient in LASSO cox regression analysis (Figure 3A,B), the SAGs signature was constructed by choosing 23 prognostic SAGs (Table 2).

**TABLE 1:** 41 cellular senescence‐related genes

Genes	Full‐names
DUSP16	Dual Specificity Phosphatase 16
EHF	ETS Homologous Factor
ITSN2	Intersectin 2
DUSP3	Dual Specificity Phosphatase 3
HDAC4	Histone Deacetylase 4
TXNIP	Thioredoxin Interacting Protein
KL	Klotho
MAP4K1	Mitogen‐Activated Protein Kinase Kinase Kinase Kinase 1
PIK3R5	Phosphoinositide‐3‐Kinase Regulatory Subunit 5
YPEL3	Yippee Like 3
CDKN2A	Cyclin Dependent Kinase Inhibitor 2A
MAP2K7	Mitogen‐Activated Protein Kinase Kinase 7
PIAS4	Protein Inhibitor Of Activated STAT 4
POU5F1	POU Class 5 Homeobox 1
EZH2	Enhancer Of Zeste 2 Polycomb Repressive Complex 2 Subunit
DGCR8	DGCR8 Microprocessor Complex Subunit
TYK2	Tyrosine Kinase 2
BTG3	BTG Anti‐Proliferation Factor 3
SOCS1	Suppressor Of Cytokine Signaling 1
G6PD	Glucose‐6‐Phosphate Dehydrogenase
TXN	Thioredoxin
DPY30	Dpy‐30 Histone Methyltransferase Complex Regulatory Subunit
AURKA	Aurora Kinase A
PDCD10	Programmed Cell Death 10
PSMD14	Proteasome 26S Subunit, Non‐ATPase 14
FXR1	FMR1 Autosomal Homolog 1
PCGF2	Polycomb Group Ring Finger 2
GAPDH	Glyceraldehyde‐3‐Phosphate Dehydrogenase
PSMB5	Proteasome 20S Subunit Beta 5
RSL1D1	Ribosomal L1 Domain Containing 1
IL1A	Interleukin 1 Alpha
CDK6	Cyclin Dependent Kinase 6
LIMA1	Lim Domain And Actin Binding 1
CAV1	Caveolin 1
SERPINE1	Serpin Family E Member 1
HSPA5	Heat Shock Protein Family A (Hsp70) Member 5
NEK6	NIMA Related Kinase 6
ASPH	Aspartate Beta‐Hydroxylase
MAP2K1	Mitogen‐Activated Protein Kinase Kinase 1
ACLY	ATP Citrate Lyase
TOP1	DNA Topoisomerase I

**TABLE 2:** Clinical characteristics of the HNSCC patients in this study

Characteristics	Number of patients	Percent (%)
Gender		
Female	133	26.65
Male	366	73.35
Age		
≤60	244	48.90
>60	255	51.10
Histologic grade		
G1	61	12.22
G2	298	59.72
G3	121	24.25
Unknown	19	3.81
T Stage		
T1	46	9.22
T2	131	26.25
T3	96	19.24
T4	171	34.27
Unknown	55	11.02
N stage		
N0	170	34.07
N1	65	13.03
N2	164	32.87
N3	7	1.40
Unknown	93	18.64
Clinical stage		
I	25	5.01
II	69	13.83
III	78	15.63
IV	259	5.19
Unknown	68	13.63
Survival status		
Dead	282	56.51
Alive	217	43.49

### 3.3 Verification of the prognostic SAGs signature for HNSCC

**FIGURE 4:**
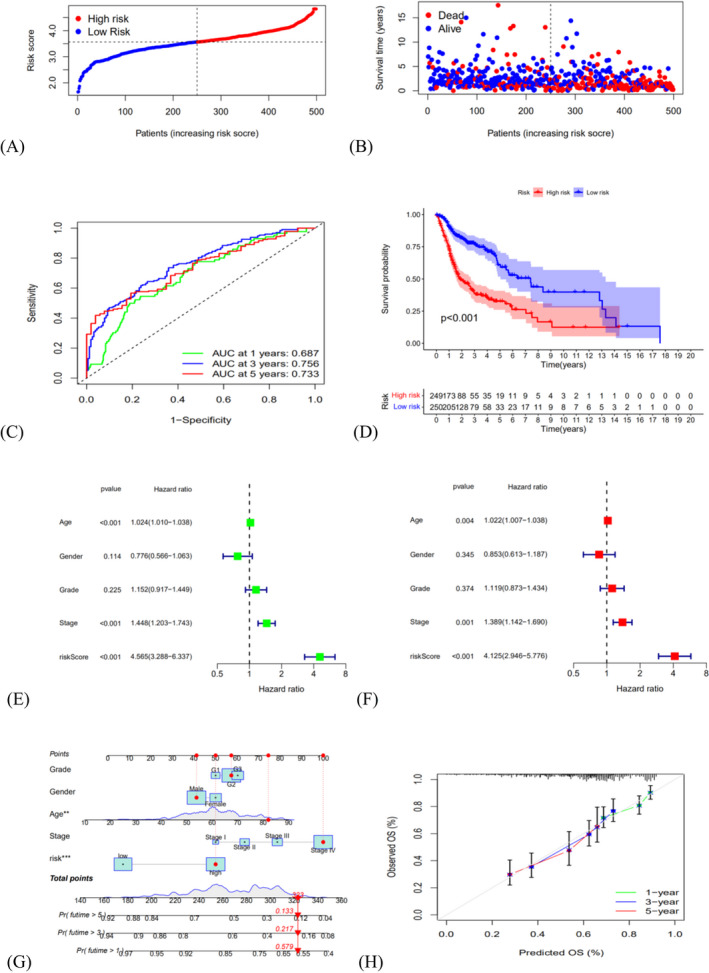
Independent prognostic value of SAGs signature in training cohorts. (A) The distribution and median value of the risk scores. (B) The distributions of overall survival status, overall survival, and risk score. (C) The AUC values of time‐dependent ROC curves for survival prediction. (D) Kaplan–Meier survival curves showing the overall survival of high‐ and low‐risk HNSCC patients divided according to the risk score (*p* < 0.001). (E) Prognostic value of the risk scores in the univariate Cox regression analysis (HR = 4.564, 95% CI: 3.287–6.337, *p* < 0.001). (F) Prognostic value of the risk scores in the multivariate Cox regression analysis (HR = 4.124, 95% CI: 2.945–5.775, *p* = <0.001). (G) The nomogram for predicting the 1‐year, 3‐year, and 5‐year overall survival. (H) Calibration plot of the nomogram for predicting 1‐year, 3‐year, and 5‐year overall survival

**FIGURE 5:**
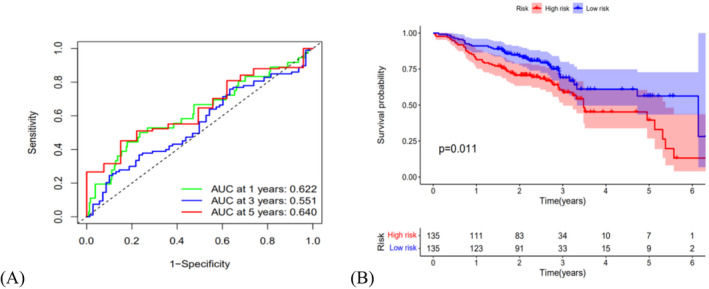
Validation of the risk model in the GEO cohort: (A) The AUC values of time‐dependent ROC curves for survival prediction of validation cohorts. (B) Kaplan–Meier survival curves of validation cohorts showing the overall survival of high‐ and low‐risk HNSCC patients divided according to the risk score (*p* = 0.011)

**FIGURE 6:**
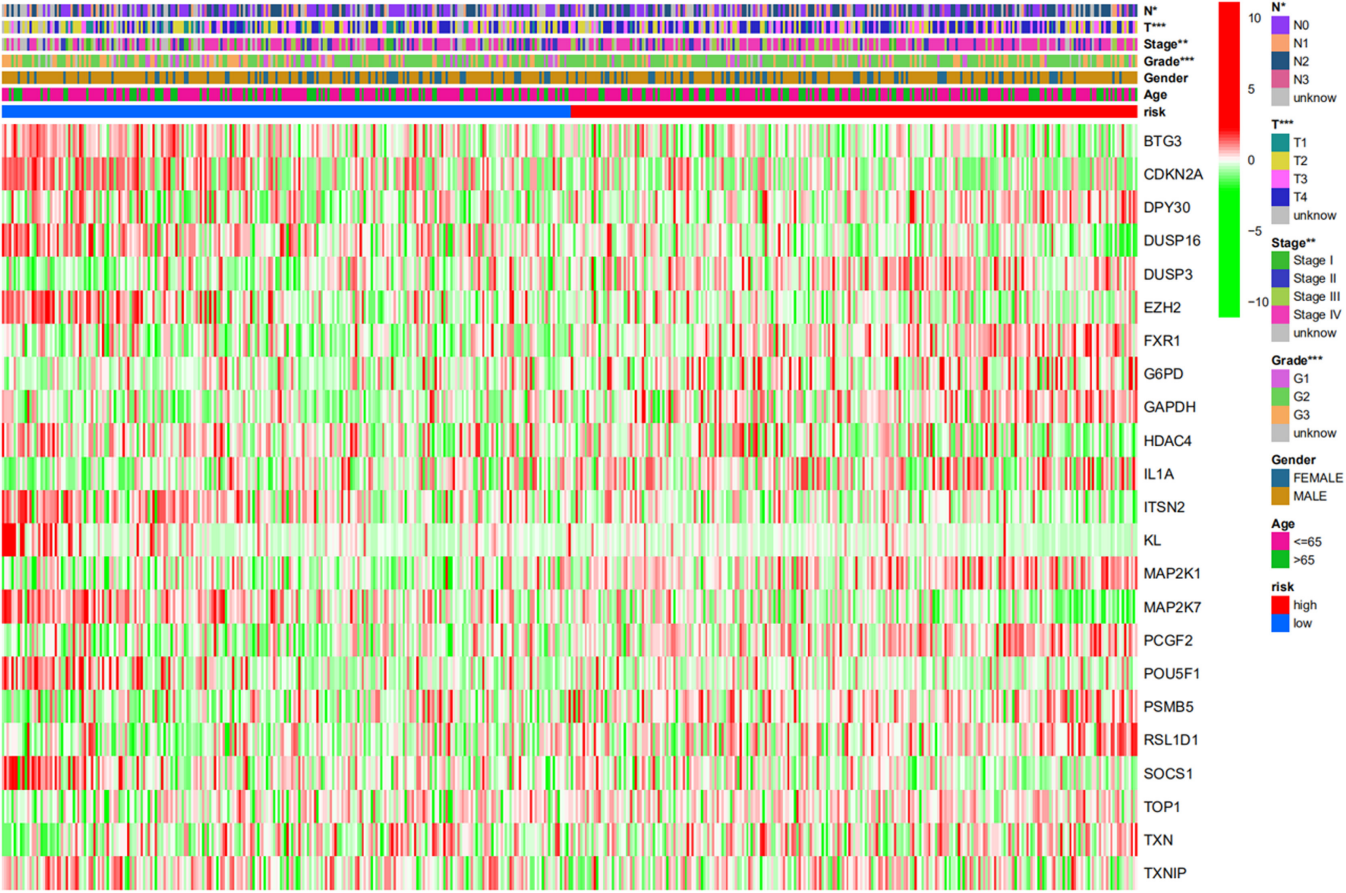
Relationship between clinical characteristics and the SAGs‐based prognostic model. A heatmap illustrated grade (***), clinical stage (**), T classification (***), and lymph node metastasis (*) were linked to the risk score

We divided 499 patients with HNSCC into the high‐senescent score group (*n* = 249) and the low‐senescent score group (*n* = 250) applying the average risk mark as the cutoff value (Figure 4A). According to Figure 4B, patients in the high‐senescent score group showed a reduced survival duration and an increased death rate than those in the low‐senescent score group. Furthermore, the area under the ROC plot was 0.687 for 1 year, 0.756 for 3 years, and 0.733 for 5 years, respectively (Figure 4C). According to Kaplan–Meier survival analysis, the high‐senescent score group showed lower survival probability than the low‐senescent score group (Figure 4D, *p* < 0.001). GSE65858 datasets as validation cohorts confirmed those results (Figure 5, *p* = 0.011). The forest plots displayed that the risk mark was a separate prognostic element of total survival for HNSCC by Univariate and multivariate Cox regression (Figure 4E; HR = 4.565, 95% CI: 3.288–6.337, *p* < 0.001, Figure 4F; HR = 4.125, 95% CI: 2.946–5.776, *p* < 0.001). We constructed a nomogram to predict survival for HNSCC patients based on tumor grade, gender, age, tumor stage, and senescent risk score. As shown in Figure 4G, by calculating the total score of each feature of some patient marked in red, we can know that his 1‐year, 3‐year and 5‐year survival rates are 57.9%, 21.7% and 13.3%, respectively. According to the calibration cures, the nomogram showed excellent prediction capacity by comparing with the ideal model (Figure 4H). Then, the association between the clinicopathological elements and risk mark was analyzed in the heatmap showed that the grade (*p* < 0.001), clinical phase (*p* < 0.01), T stage (*p* < 0.001), and lymph node metastasis (*p* < 0.05) were greatly related to the risk score (Figure 6).

### 3.4 Functional enrichment analysis

**FIGURE 7:**
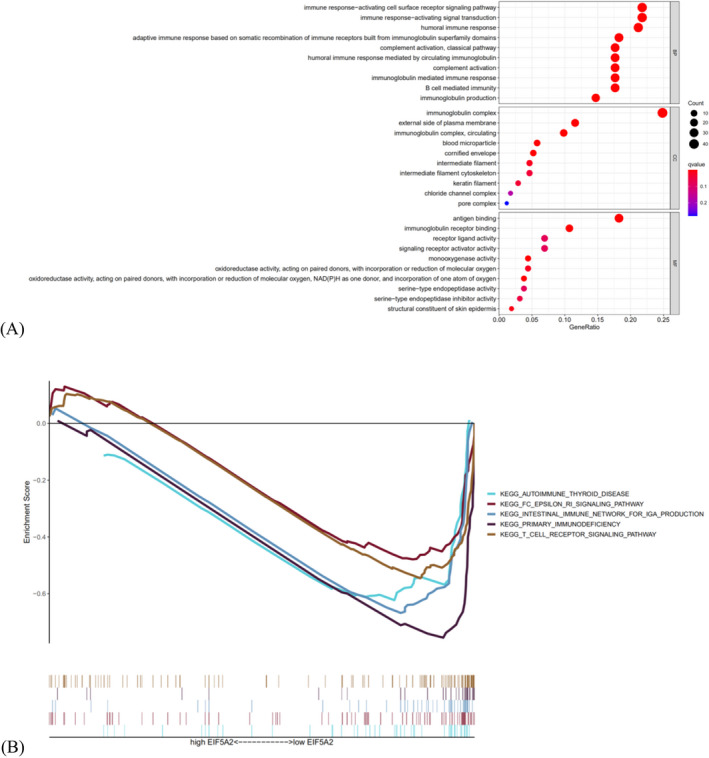
Functional enrichment analysis. (A) The top ten biological process terms, cellular components terms, molecular functions terms of GO analysis. (B) GSEA analysis showing five pathways enriched in the low‐risk group

According to Figure 7A, the results of GO and GSEA analysis indicated that the DEGs were mainly participated in the cellular or humoral immune response, complement activation, and immunoglobulin production. Uniformly, some immune‐associated paths were greatly increased in the low‐risk group, such as T‐cell receptor signaling path, autoimmune thyroid disease, intestinal IgA generation and Fc epsilon Ri signaling path (Figure 7B).

### 3.5 The association between the SAGs signature and immune state

**FIGURE 8:**
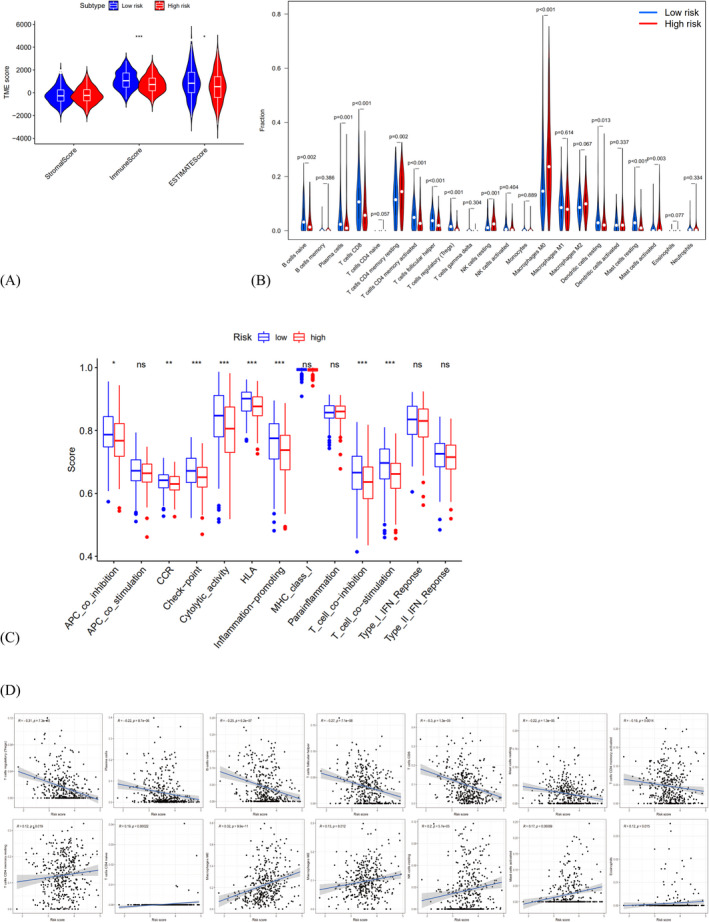
Effects of SAGs‐based prognostic model on immune cell infiltration. (A) Comparison of the immune, stromal, and estimate scores in the low‐ and high‐risk groups, respectively. (B) The violin plot of different infiltration levels of immune cells between high‐ and low‐risk patients. (C) The violin plot of different infiltration levels of immune‐related functions between high‐ and low‐risk patients. (D) The correlation of risk score and immune cells infiltration. Adjusted *p*‐values were showed as: ns, not significant; *p < 0.05; **p < 0.01; ***p < 0.001

The tumor immune microenvironment of HNSCC was further evaluated. HNSCC patients in the low‐senescent score group showed higher immune marks and estimate marks than the high‐senescent score group (Figure 8A), and there is no statistical significance only for the stromal score. Based on these observations, the low‐senescent score group may recruits more inflammatory cells. Hence, the situation of immune cells infiltration of HNSCC was analyzed. According to expectation, naive B cells, plasma cells, T cells follicular helper, CD8+ T cells, activated T cells CD4 memory, T cells regulatory, remaining dendritic cells and mast cells were upregulated in the low‐senescent score group, and with lower infiltration of resting T cells CD4 memory, resting NK cells, M0 macrophages and activated mast cells (Figure 8B). The ssGSEA analysis indicated high activation of checkpoint, cytolytic activity, HLA, inflammation‐promotion, T cell co‐inhibition and T‐cell co‐stimulation immune pathways in low‐senescent score group (Figure 8C). Finally, association analysis showed that T cells regulatory, plasma cells, naive B cells, T cells follicular helper, CD8+ T cells, resting mast cells and activated T cells CD4 memory were strongly negatively correlated with risk marks. Resting T cells CD4 memory, naive T cells CD4, M0 macrophages, M2 macrophages, resting NK cells, activated mast cells and eosinophils were positively related to risk mark (Figure 8D).

### 3.6 Immunotherapy forecast of SAG signature for HNSCC

**FIGURE 9:**
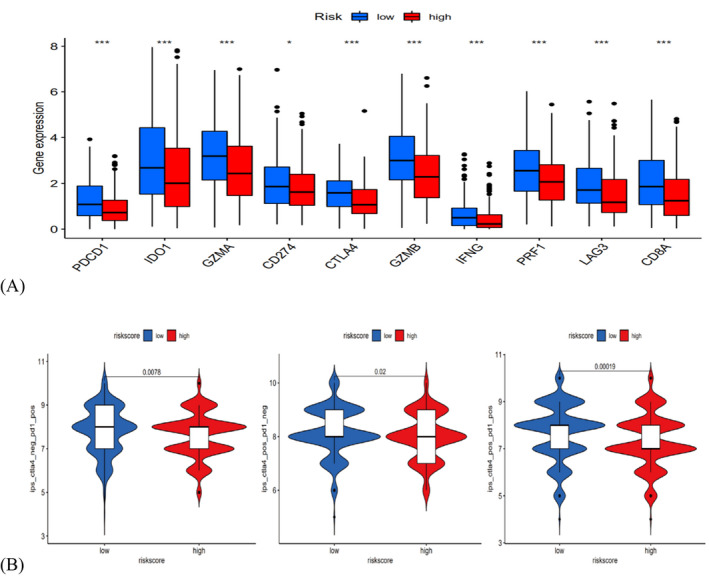
Immunotherapy prediction of SAGs‐based prognostic model for HNSCC. (A) Immune checkpoint inhibitors related genes expression between high‐ and low‐risk patients. (B) Differences in immunophenoscores between patients in high‐ and low‐risk groups received anti‐PD1 alone, anti‐CTLA4 alone, and combination therapy with anti‐CTLA4 and anti‐PD1

For researching the effect of immunotherapy, the relationship between risk marks and Immune checkpoint inhibitors (ICI) associated genes were analyzed. The expression level of ICI associated genes in the low‐risk group were mostly greatly higher than the high‐risk group (Figure 9A, *p* < 0.001), indicating that the low‐risk HNSCC patients better responded to ICI. This guess was confirmed with TCIA and revealed that the low‐senescent score group better responded to PD‐1 inhibiting agent alone (*p* = 0.0078), CTLA4 inhibiting agent alone (*p* = 0.02) or an integration of PD‐1 and CTLA4 inhibiting agent (*p* = 0.0019) (Figure 9B).

### 3.7 Chemosensitivity analysis

**FIGURE 10:**
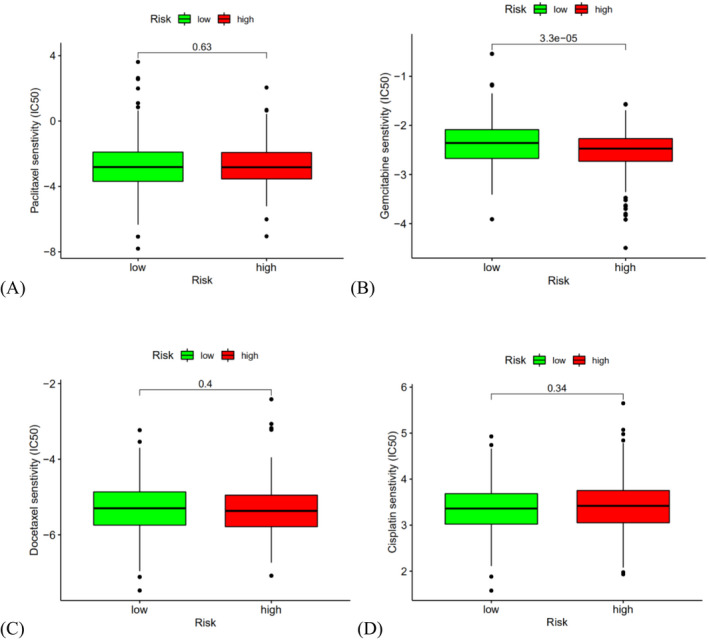
Drug sensitivity analysis of (A) paclitaxel, (B) gemcitabine, (C) docetaxel, and (D) cisplatin for HNSCC patients at the high‐ and low‐risk cohorts. The IC50 values of gemcitabine (B) decreased significantly in the high‐risk group; The IC50 values of paclitaxel (A), docetaxel (C) and cisplatin (D) were not significantly different between the high‐ and low‐risk cohorts

By analyzing the IC50 values of four common chemotherapeutic drugs for HNSCC patients, we found that the IC50 value of gemcitabine (Figure 10B, *p* < 0.001) was significantly reduced in the high‐risk group, suggesting that patients at high risk of aging had a better response to the chemotherapy drug; However, IC50 values of paclitaxel (Figure 10A), docetaxel (Figure 10C), and cisplatin (Figure 10D) did not differ significantly between high‐ and low‐senescent score groups.

## 4 DISCUSSION

As the sixth most frequent cancer globally, head and neck squamous cell carcinoma (HNSCC) is driven by abnormal changes of genetic, epigenetic, and environmental elements.^11^ To promote the overall survival of HNSCC, reliable and novel promising biomarkers are urgent requirements for HNSCC patients nowadays. Cellular senescence means a stress response with a permanent cell cycle arrest (Figure 1A). There are many effector mechanisms of senescence recognized and characterized particularly in the p53 and p16/Rb pathways triggered by persist DNA damage response (pDDR).^12^ The pDDR positively regulates the SASP, which can be enlarged with positive feedback loops between proinflammatory cytokines and the transcription elements (NF‐kB and C/EBPb). However, p53 negatively modulates the SASP. The SASP enhances senescence by autocrine activities. Extracellular matrix, normal cells, tumor cells, and immune cells are the objects of paracrine activities of the SASP. Senescent cells can be removed by immune cells.^4^ It is also possible that the SASP plays systemic role in tissue remodeling and immune microenvironment^13^, ^14^. Hence, cellular senescence might exert a basic effect on the occurrence and development of HNSCC. We identified novel senescence‐associated genes (SAGs) differentially expressed in HNSCC and formed a reliable SAGs‐based signature to forecast prognosis with sufficient performance by an integrative bioinformatics.

This research set up a prognostic signature including 23 SAGs (BTG3, CDKN2A, DPY30, DUSP16, DUSP3, EZH2, FXR1, G6PD, GAPDH, HDAC4, IL1A, ITSN2, KL, MAP2K1, MAP2K7, PCGF2, POU5F1, PSMB5, RSL1D1, SOCS1, TOP1, TXN and TXNIP) finally. It is worth noting that CDKN2A is a Protein Coding gene. Diseases related to CDKN2A include Cutaneous Malignant Melanoma.^15^, ^16^, ^17^, ^18^ Among its associated pathways are DNA Damage Response and Mitotic G1‐G1/S phases.^19^ IL1A (Interleukin 1 Alpha) is related to cytokine activity and interleukin‐1 receptor binding and its associated pathways are IL1α‐NF‐κB pathway and cytokine signaling in Immune system.^20^, ^21^, ^22^ As shown Figure 1A, those two genes (CDKN2A and IL1A) are closely associated with the progress of cell senescence. MAP2K1 is Mitogen‐Activated Protein Kinase Kinase 1, which is related to pathways of apoptosis and survival anti‐apoptoticaction^23^, ^24^. Besides, anther genes of SAGs like DUSP16、KL、PSMB5 and SOCS1 are involved in Cytokine Signaling in immune system according to gene database.^25^, ^26^, ^27^, ^28^, ^29^, ^30^

In brief, our findings suggest that these 23 prognostic SAGs signature significantly influence the survival time of cancer patients, which might offer hidden therapeutic objects for HNSCC. The results of survival analyses and univariate or multivariate Cox regression analyses show that the SAGs signature is an accurate predicting agent of outcome in HNSCC. Therefore, we constructed a nomogram containing multiple factors such as senescent risk score and age to accurately predict survival in HNSCC patients. The calibration curve verifies the accuracy of the model. Large sample studies will help test this projection in future. In addition, the association between the clinical elements and risk mark has been assessed. The grade (*p* < 0.001), clinical phase (*p* < 0.01), T stage (*p* < 0.001), and lymph node metastasis (*p* < 0.05) were greatly linked to the risk score.

To reveal the biological roles of SAGs signature, GO improvement analysis and GSEA analysis were made, showing that immune‐associated biological processes and pathways were increased in the low‐senescent score group, which recruited more functional B or T cells.^31^, ^32^, ^33^, ^34^ Jiang and Zeng et al. found that the survival rate of patients with melanoma is associated with immune invasion. They established an aging risk model to identify and predict the immune microenvironment of malignant melanoma. Studies indicated that immunosuppressive cytokines were upregulated in patients with malignant melanoma at low risk of aging, which is consistent with our study^35^, ^36^. In contrast, the high‐senescent score group showed higher quantity of M2 macrophage cells, which was positively related to the risk mark. The research found that M2 macrophages are immune suppressive cells associated with angiogenesis and tissue remodeling, which enhance the immune suppression ability of tumors to promote cancer.^37^ We summarize that cell senescence have systemic effects on tissue remodeling and immune microenvironment, which may mediate the pathogenesis of HNSCC.

Current studies found that immunotherapy like pembrolizumab and nivolumab have been useful in HNSCC, inducing the human immune system to eliminate cancer.^11^ In our study, we found that ICI associated genes (PD‐L1, CTLA4, PD1) were greatly improved in the low‐senescent score group, which had a better response to PD‐1 inhibiting agent or CTLA4 inhibiting agent or the integration of PD‐1 and CTLA4 inhibiting agent with TCIA.^38^ We conclude that our SAGs signature might make contributions to developing immunotherapy measures for HNSCC patients.

Chemotherapy is one of the important treatment methods for advanced head and neck squamous cell carcinoma, and its clinical efficacy is worthy of affirmation. Some advanced patients will have significantly reduced tumor size after chemotherapy, and will get the opportunity for surgery again. Gadhikar et al. reported that cisplatin inhibits cell growth by inducing senescence of head and neck cancer cells.^39^ Therefore, aging genes may be associated with chemotherapy resistance of squamous cell carcinoma of the head and neck. In order to further obtain the predictive value of aging genes in chemotherapy response of HNSCC patients, we calculated the sensitivity of four commonly used chemotherapy drugs to HNSCC patients according to the “pRRophetic” algorithm. Interestingly, our study found that gemcitabine was more sensitive in patients with high‐senescent risk, which provides a priority reference value for rational clinical selection of chemotherapy agents for HNSCC patients.

There are several limitations in this research. The conclusions would be more useful in case of experimental verification. On basis of the research outcomes, new research will perform HNSCC animal models to dig the association of 23 senescence‐associated genes expression and immune microenvironment.

## 5 CONCLUSIONS

A new 23 SAGs signature was established for predicting outcomes of HNSCC. SAGs potentially influences tissue remodeling and immune microenvironment for the occurrence and development of HNSCC. Our outcomes offer insight to forecast results and recognize therapeutic targets for HNSCC patients.

## CONFLICT OF INTEREST

The author has declared that there is no competing interest associated with this study.

## Data Availability

The data used for our analysis in this study are openly available at (https://portal.gdc.cancer.gov/ and http://www.ncbi.nlm.nih.gov/geo/).
